# A Theoretical Study of the Hidden Wounds of War: Disenfranchised Grief and the Impact on Nursing Practice

**DOI:** 10.5402/2011/954081

**Published:** 2011-04-18

**Authors:** Janice A. Aloi

**Affiliations:** School of Nursing, University of Medicine and Dentistry, 65 Bergen Street, Room 1011, P.O. Box 1709, Newark, NJ 07101, USA

## Abstract

Combat veterans face enormous challenges upon the return to civilian life, one of which is the ability to integrate incidences of death and killing into a healthy postdeployment life. This paper presents the lived experience of grief and loss resulting from the trauma of war. Social constructionist theory, due to its emphasis on meaning-making, serves as the theoretical framework. The effects of inhibited mourning due to the inability to mourn in combat and lack of nurturing upon returning home are described. Personal excerpts derived from interviews of warfare from veterans that experienced death and killing are presented. It is suggested that combat veterans experience a unique form of grief and therefore require a style of grieving that differs from those that have not served on the battlefield. Regardless of the point of care, nurses are positioned to help with the challenges of readjustment. A better understanding of combat veterans as a disenfranchised group would enable nurses to intervene in ways that contribute to the readjustment process.

## 1. Introduction

Serving in a combat zone has many life-long consequences, one of which is the intense, complicated grief on the part of the veteran. For many years to come, nurses practicing in all health care settings will be providing care to veterans suffering not only from the physical, but also the emotional challenges of readjustment to civilian life. Postwar emotional adjustment problems are poised to be a considerable health risk for Iraq and Afghanistan veterans. There is agreement in the literature that grief and loss play a major role in the veteran's return to civilian life [1–4]. More than 100,000 combat veterans sought help for emotional problems since the start of the war in Afghanistan, according to the United States Department of Veterans Affairs [5]. This number points to the importance of intervention to help veterans resolve the feelings of loss surrounding personal injuries and the violent deaths of fellow soldiers. The role that nurses play can be critical in determining the aftermath of the life-altering experience of combat. That role exists far beyond treating the physical wounds of war which, for many, will result in frequent and possibly life-long visits to a wide variety of health care settings. There is considerable documentation that the loss of comrades during combat is a significant source of distress [1–4]. Lewis [1] claims that combat veterans experience “elevated symptoms of complicated mourning,” a unique form of grief that adds to the challenge of successful adjustment to postwar life. According to Doka [4], veterans are one of several populations that have “little or no opportunity to mourn publicly.” Empirical studies have not focused on disenfranchised or unresolved grief as one possible result of combat experience. Doka [4] defines disenfranchised grief as “grief that is not openly acknowledged, socially accepted, or publicly mourned.” For the most part, the combat veteran experience of loss has been unheard and unacknowledged by family and society, despite its profound lasting effects. The recognition, support, acknowledgement, and interventions utilized within the context of the nurse-client relationship can be key ingredients for effective resolution. Unfortunately, many nurses do not recognize the unresolved grief that many veterans carry with them throughout a lifetime, thus contributing to their disenfranchisement. Nurses in all settings will be interfaced with the ever-increasing number of combat veterans and, thus, will be in position to acknowledge the disenfranchisement, explain the reasons for it, and suggest interventions that address the depth and power of losses occurred in battle.

## 2. Grief and Mourning

Stories of warfare provide testimony to the challenge faced by any veteran upon his or her return to civilian life. For those who have experienced death and killing, the challenge may present major problems. In her paper “Grief, Trauma and Combat,” Lewis [1] offered two reasons for veterans' grief: (1) “the death of fellow soldiers and the loss of their own assumptive world” and (2) “sanctified and rewarded killing.” She further points out that despite being a society with a long history of wars, “veterans have not always received adequate nurture when they have returned home” resulting in the inability to move through the stages of grief, which many know as denial, anger, bargaining, depression, and acceptance [6].

Kubler-Ross [6], in her seminal work on death and dying, revealed that when people are dying or grieving for a personal loss, he or she will proceed through a series of emotional stages in an effort to resolve the loss. Briefly, the five stages are as follows.

- Stage One—Denial: “No, this can't be happening to me.” The loss is not accepted or, perhaps, not even recognized.

(ii) Stage Two—Anger: “Why me?” Feelings of persecution and anger towards others are felt.

(iii) Stage Three—Bargaining: “I promise I will…” Begging, wishing, and attempts to make a deal are made.

(iv) Stage Four—Depression: “I don't want to live anymore…” Feelings of hopelessness, frustration, bitterness and self-pity are experienced.

(v) Stage Five—Acceptance: “I have to go on…” Painful feelings are less frequent and less intense. Comfort and healing occur.

Kubler-Ross suggests that true healing can only occur when these five stages are experienced by the griever. She further states that problems arise when the emotions of anger, fear, sadness, jealousy, and love are distorted and, therefore, difficult to experience fully or not at all. According to Grossman [2], “at some level every psychologically healthy human being who has engaged in or supported killing activities believes that his action was “wrong” and “bad,” and he must spend years rationalizing and accepting his actions.” Grossman claimed that some combat veterans who have killed cannot proceed past Kubler-Ross' first stage of the grieving process and so never achieve a state of acceptance. He argues that Kubler-Ross' prescription for grieving does not adequately address the complex needs felt by veterans as they return from combat. It must be noted, however, that Kubler-Ross' paradigm was conceived in a totally different situational and historical context.

When a veteran who has worked through his or her grief is asked “Did it bother you?” the answer is likely to be, “Hell yes… You can't go through that without being influenced.” In contrast, Grossman [2] says, the veteran who has repressed and denied his emotions would say, “No, it never really bothered me… You get used to it.” The ability to move through the denial stage appears to be especially challenging for combat veterans who are one of the many populations in which a sense of loss is experienced, but the social constructs may not be in place to facilitate the healing process. Anyone who is grieving needs support for the reality of the loss and the validity of their grief. If the members of the veteran's social network are unable to recognize the loss or provide the necessary support, the returning veteran will not be able to validate his or her grief. This may, in turn, lead to life-long adjustment problems. Many veterans were told that they will forget, and yet, few veterans are able to forget Flinn [7]. 

Worden [8], in his book *Grief Counseling and Grief Therapy*, presents a model of grief counseling which includes four aspects that he thinks are necessary for successful grief resolution. First, the reality of the loss must be accepted. Second, the pain of grief must be experienced. Third, the grieving person must adjust to the environment from which the lost person or object is missing. And, fourth, emotional energy must be withdrawn from the lost person or object and reinvested in someone or something else. Lewis points out that many Vietnam veterans, who are now of age 55 and older, are seeking out counseling regarding war-related issues for the first time. These men and women, she claims, are suffering the effects of inhibited mourning due to the inability to mourn in combat, together with the lack of adequate nurturing upon returning home from war. Many have not even achieved the first step in Worden's model of grief counseling. Nathaniel Ganzeveld is an Iraq War veteran whose adjustment difficulties occurred well after his return home:

My symptoms didn't show up right away. Then everything just caught up to me and hit me all at once. Overseas there is no time to be scared and frightened, but you experience all this high stress. Then you get home, you relax, and then it comes rushing up. I have nightmares that I'm back in Iraq watching my dear friend get killed [9].

A trip to the Vietnam Memorial Wall in Washington D.C., according to Lewis [1], can help veterans such as Nathaniel and others who never moved beyond the first stages of grief, to facilitate the process of recognizing the loss in an effort to begin the work of mourning.

The prescription for war is to kill the enemy, which is the most difficult aspect of war for veterans to process upon returning home [1]. Grossman [2], in *On Killing, *argues that the grief of a soldier who has witnessed or who has taken part in killing is unique and does not, nor can it, follow the traditional prescription for grief and mourning. The soldier's grief, according to Grossman, is significantly different when it involves killing human beings. He describes the process as similar to that of Kubler-Ross, but having a much greater magnitude and intensity than is found in her five stages. 

The goal of successful grief resolution involves reaching a point at which one is “neither depressed nor angry about his fate” [6]. The disenfranchised grief experienced by many combat veterans requires a sensitive and knowledgeable approach by nurses, families, and the health care system for this outcome to occur.

## 3. Disenfranchised Grief

Doka and Worden, who have written extensively on disenfranchised grief, discussed the role that grieving plays in the postwar adjustment process. They both argued that veterans are one segment of a disenfranchised population suffering from the effects of loss. In Doka's [4] book *Disenfranchised Grief: New Directions, Challenges and Strategies for Practice*, he offered reasons for the occurrence of disenfranchised grief, all of which have implications for veterans. Doka [4] defined disenfranchised grief as “grief that persons experience when they incur a loss that is not or cannot be openly acknowledged, publicly mourned, or socially supported.” The inability to mourn in combat, as described by Lewis [1], has been cited as a key factor in instances of disenfranchised grief. 

Doka [4] offered three reasons for the occurrence of disenfranchised grief, all of which have implications for combat veterans. First, when the relationship between the griever and the lost person is not recognized, society does not expect the loss to produce much of a reaction. In our society, discussions of grief primarily center on family members and close friends. Grief may be disenfranchised in war situations in which the relationship between the deceased and the survivor is not based on established ties. John Early, a veteran described the strength of bonds formed on the battlefield in this way:

This is going to sound really strange, but there is a love relationship that is nurtured in combat because the man next to you—you're depending on him for the most important thing you have, your life, and if he lets you down you're either maimed or killed. If you make a mistake the same thing happens to him, so the bond of trust has to be extremely close, and I'd say this bond is stronger than almost anything, with the exception of parent and child. It's a hell of a lot stronger than man and wife—your life is in his hands, you trust that person with the most valuable thing you have [2, page 90].

Next, Doka [4] stated that disenfranchised grief occurs when the loss is not recognized or socially validated. This World War II veteran speaks of loss that, at the time of his homecoming, he considered socially insignificant, “It didn't hit me all that much then, but when I think of it now—I slaughtered those people. I murdered them [2,page 88]”.

Doka [4] describes the third reason for the occurrence of disenfranchised grief as one in which the griever is considered incapable of grief and cites the young and the very old as examples, despite evidence to the contrary. In this situation, the griever is not recognized due to his or her lack of the ability to grieve. Here, a veteran describes the efforts to desensitize soldiers to the act of killing,

“We'd run PT (physical training) in the morning and every time your left foot hit the deck you'd have to chant ‘kill, kill, kill, kill.' It was drilled into your mind so much that it seemed like when it actually came down to it, it didn't bother you, you know [2, page 250]?”

Doka [4] added two additional categories to the three that he initially identified: (1) circumstances of the death, and (2) ways individuals grieve. He pointed out that the nature of the death might discourage the griever from seeking support from others as well as diminishing the willingness or interest of others in providing support. Vietnam veterans have reported cases in which support was withheld punitively, such as the following:

There was a bias toward Vietnam veterans, especially after the My Lai massacre broke... clinicians reflected the ambivalence about the war, I mean, they really weren't any different than the regular population. The majority of clinicians at the VA were in one way or another very intimately connected with World War II… They had this sort of noble view of World War II as a glorious fight against satanic enemies and that the World War II veterans were as pure as the driven snow… (Also) we had antiwar clinicians in my agency who didn't want to talk with Vietnam veterans because they were baby killers who should have known better and not have gone in the first place [10].

It has been suggested by Doka [4] that there are different styles of grieving. 

Expressions of grief that are more physical, cognitive, behavioral, or that fall beyond the societal rules can be disenfranchising by nurses, hospital personnel, counselors, and the larger community.

## 4. The Effects of Disenfranchised Grief

 Many of the difficulties experienced by veterans following homecoming are expressions of disenfranchised grief. The thoughts, feelings, and experiences described by veterans resonate with the concepts put forth by Doka [4]. He calls the problems paradoxical: “The very nature of disenfranchised grief creates additional problems for grievers while removing or minimizing their sources of support [4].” 

The emotions associated with grief are intensified and complicated when grief is disenfranchised [4]. Many veterans believe that they are undeserving and inherently bad people, resulting in serious self-esteem issues. Some feel punished for their actions. It has been noted by Doka [4] that people with disenfranchised grief often have difficulty coping with subsequent losses. The pattern tends to repeat itself resulting in further disenfranchisement and unhealthy coping mechanisms. For many combat veterans, the consequence of battle was the first major loss in their lives. Attempts at resolution of further losses will be insufficient or inappropriate for true healing of grief to occur.

It had been noted by several researchers that in many cases of disenfranchised grief there are no funeral rituals [2, 4, 11]. Grief such as in divorces, abortions, the loss of a pet, and certainly the pain and remorse felt by combat veterans may all lack the sense of closure that a funeral or some other ritual may offer. Grossman noted that throughout history, tribes, societies, and nations have provided returning warriors with various types of purification rituals. Rituals, he said, provide the veteran with a way of working through the stress, grief, and guilt of the killing experience. The power of the purification ritual is articulated in the following excerpt:

Societies have always recognized that war changes men, that they are not the same after they return. That is why primitive societies often require soldiers to perform purification rites before allowing them to rejoin their communities. These rites often involved washing or other forms of ceremonial cleansing. Psychologically, these rituals provided soldiers with a way of treating guilt by providing a mechanism through which fighting men could decompress and relive their terror without feeling weak or exposed. Finally, it was a way of telling the soldier that what he did was right and that the community for which he fought was grateful and that, above all, his community of sane and normal men welcomed him back [2, page 272].

Following World War II, soldiers spent days debriefing as they sailed home, upon which they were met with parades and other tributes in their communities. Grossman [2, page 274] cites parades as “an essential rite of passage to the returning veteran in the same way that bar mitzvahs, confirmations, graduations, weddings, and other public ceremonies are to other individuals at key periods in their lives. Memorials and monuments mean to the grieving veteran what funerals and tombstones do to any bereaved loved one. Our current society needs to head this warning and be reminded that the Vietnam Veterans Memorial came twenty years too late [12].”

Lewis [1] maintains that veterans are expected to mourn for family and friends, but not for those who have been killed in combat. Veterans may also grieve for themselves. The loss of innocence, youthful idealism, former self-concept, and other areas of personal growth might require processing and reconciliation. When people, she argues, are not recognized as grievers, they will not be given the support needed to move through the grief process, resulting in emotional casualties and continued disenfranchisement.

## 5. Resolution of Disenfranchised Grief

Many combat veterans upon returning home report mood disturbances and expressions of grief following their initial feeling of overwhelming exhilaration and thankfulness at having survived. It was noted by Milliken [10] that adjustment issues become evident after the initial euphoria of homecoming subsides. Perhaps the euphoria upon homecoming prevents the emergence of negative feelings, and only after the euphoria fades, do the psychological problems of postwar adjustment become prominent. Doka [4] points out that because the problems are so deep-seated, healing is difficult, but not impossible. Veterans must first and foremost acknowledge and validate the loss in order to work through the grief process. It is critical that nurses be sensitive to this need when caring for veterans, even if the veteran's war experience occurred well in the past. The nurse's overall goal for the client who is a combat veteran should be the promotion of dialogue. Ideally, the nursing staff should be aware of the veteran's status regarding his or her return to civilian life. Nurses may be afraid to bring up the subject of war due to their own discomfort or negative attitude about the war effort. A self-assessment on the part of the nurse will help the nurse to create an understanding and accepting climate in which the veteran's thoughts and feelings can be expressed. 

The loss of fellow soldiers and innocent civilians is often cited as a major reason for the feelings of depression upon homecoming, regardless of the participants' war affiliation [4, 8]. Also significant are the feelings of intense guilt for surviving intact when others did not or disgust about those killed or maimed, including those across enemy lines. A preoccupation with the fate of fellow comrades that were whisked away following an injury on the battlefield is a contributing factor to the veteran's feelings of loss. Doka [4] referred to this “lack of closure” as a significant barrier in the adjustment process. He reminds us that wartime relationships are generally not recognized and, therefore, their loss is not expected to produce much of a reaction. This notion is in direct contrast to the value of wartime relationships felt by veterans as expressed in the motto “No soldier left behind.” Nurses may mistakenly think the veteran does not want to discuss the losses experienced on the battlefield and thus be unsure about how to treat the veteran's emotional needs. This can foster an avoidant approach by the nurse resulting in the lack of a quality interaction and a focus on the physical aspects of nursing care. An avoidant approach by the nursing staff can contribute to further disenfranchisement. The nurse may gently bring up the subject in order to display a willingness to talk and foster an exchange. Listening, without giving advice or one's own opinion regarding the worthiness of the current wars, is needed by combat veterans, often more than the need for physical care. The nurse's use of recognition and validation of the losses that occur in a war zone experience can help the veteran move toward effective resolution by allowing him or her to grieve.

The resolution of disenfranchised grief following combat entails the integration of the combat experience into every other aspect of the veteran's life. Family, work, friendships, and community all contain elements that can influence the resolution of grief. The nurse is in a key position to educate the family on the dynamics of disenfranchised grief and the role it plays in the combat veteran's postwar adjustment to civilian life. The time that the nurse spends with the client who is a veteran is short, however, providing the family with tools to help their loved one mourn the loss of a body part, physical function, fellow comrades, or innocent civilians can make the difference between a lifetime of disenfranchisement and successful resolution of the grieving process. The following family interventions are suggested by the author. They include

- providing education at the family's level of understanding,
- allowing divergent grief responses,
- resisting the urge to control or condemn the veteran's grief responses,
- acknowledging the importance of the loss,
- affirming the value of the war effort,
- validating that the veteran has undergone a life-altering experience,
- celebrating the veteran's homecoming,
- accepting that he or she is a changed person,
- planning a memorial service to allow the veteran to mourn his or sense of loss,
- visiting a war memorial or war museum,
- encouraging the veteran to join a veteran's organization for peer support,
- laying wreaths on Memorial Day and Veteran's Day as a way of acknowledging the sacrifice,
- creating a memory board, scrapbook or photo album to confirm the reality of experience.

## 6. Conclusion

Alterations in mood, grief, and loss play a major role in readjustment to civilian life following a combat experience. Loss is a well-known psychological theme of combat. Not well-known is just how vulnerable this population is. The 100,000 plus combat veterans that sought help for emotional problems since the start of the war in Afghanistan provide testimony that the emotional issues that veterans struggle with have not been definitively described or adequately addressed. Combat veterans experience a unique form of grief which empirical studies have not focused on. The absence of recognition by nurses, hospital personnel, and society can contribute to the disenfranchised grief experienced by many veterans. The integration of the combat experience into every other aspect of the veteran's life is essential for successful readjustment to occur. It is very important for nurses to understand the psychological effects of war because of their possible interference with the nurse's ability to support the returning veteran and provide the needed therapeutic outlets. Effective nursing intervention with a focus on educating family members on the veteran's need for open expression, different ways of expressing grief, and the provision of specific strategies to provide the needed support can contribute to the healing process necessary to resolve the often overwhelming feelings of loss and guilt felt by returning soldiers.
